# Mentoring Experiences of Aging and Disability Rehabilitation Researchers

**DOI:** 10.1155/2010/491368

**Published:** 2010-07-04

**Authors:** Mary Egan, Kerry Byrne, Paul Stolee, Judy King

**Affiliations:** ^1^Élisabeth Bruyère Research Institute, 43 Bruyère Street, Ottawa, ON, Canada K1N 5C8; ^2^School of Rehabilitation Sciences, University of Ottawa, 451 Smyth Road, Ottawa, ON, Canada K1H 8M5; ^3^Department of Sociology, University of British Columbia, Vancouver, BC, Canada V6T 1Z1; ^4^Department of Health Studies and Gerontology, University of Waterloo, 200 University Avenue West, Waterloo, ON, Canada N2L 3G1

## Abstract

*Objectives*. To explore research mentoring experiences and perceived mentoring needs of aging and disability researchers at different career stages. *Design*. Focus group and individual interviews with rehabilitation researchers at various career stages based in hospitals, universities, and hospital-based research institutes in Ontario, Canada. *Results*. The overall theme was *mentoring for transition*. Participants across career stages referred to helpful mentoring experiences as those that assisted them to move from their previous stage into the present stage or from the present stage into their next career progression. Unhelpful mentoring experiences were characterized by mentor actions that were potentially detrimental to transition. Subsumed under this theme were three categories. The first, “hidden information” referred to practical information that was difficult to access. The second “delicate issues” referred to helping the participant work through issues related to sensitive matters, the discussion of which could put the participants or their colleagues in a vulnerable position. The third category was “special challenges of clinician-researchers”. *Conclusions*. Helpful mentoring for rehabilitation researchers working on concerns related to aging and disability appears to be characterized by interaction with more experienced individuals who aid the researcher work through issues related to career transition.

## 1. Introduction

Mentorship has been defined as, “a partnership in personal and professional growth and development” [1, page 1103]. Adequate mentorship is seen as a key factor in the retention and development of rehabilitation researchers and has been identified as a top priority for the field [2]. Mentoring relationships are associated with increased research productivity among both junior and senior investigators [1, 3, 4]. Mentoring programs for individuals at all levels of career development may be particularly important for female rehabilitation researchers, who rate their skills and readiness to carry out research and advance to leadership positions as less developed than their male colleagues, while shouldering greater domestic responsibility [5]. 

In reviewing mentorship functions within academia in general, Mullen and Hutinger [6] describe two groups of mentoring activities. Career activities include sponsorship, coaching, protection, exposure, visibility, and challenging work assignments, as well as transmission of the professional culture. Psychosocial-related activities include emotional support, role-modeling, acceptance and confirmation, counselling, and, possibly, friendship. 

De Janasz and Sullivan [7] summarize the objective of mentoring within academia as assisting the mentee to develop knowledge related to “why” (what is driving the career), “how” (specific skills related to research), and “whom” (development of webs of relationship necessary for project completion), with the content related to each of these categories changing over the course of an academic career. They further note that mentoring in academia is not as developed as it is in industry due to different reporting relationships, the perception that many of the skills required to succeed have already been learned throughout the long training process, and the tendency of some academic departments to test new recruits by watching if they will “sink or swim”. 

Recently, however, there seems to be an emergence of interest in mentoring within academia, possibly related to an escalation in expectations for research productivity at many institutions [8, 9]. Several types of faculty mentoring programs are currently described in the literature. These include traditional dyadic or grooming mentoring programs (one junior faculty member paired with one senior professor), as well as less traditional approaches. These include mentoring circles (one mentor working with a group of several mentees) [10], triangular mentoring (each mentee has two mentors: one from his or her own department and one from the institution at large) [6], external mentoring (the mentee is assigned a mentor within his or her own research area but from another institution [8], strategic collaboration (a small group of pre-tenure and newly tenured faculty is paired with two senior professors) [9], and peer-mentoring (mentees meet together to provide each other with support and direction), with [11, 12] or without [13] an assigned facilitator. 

As noted above, mentoring has also been seen as critical for rehabilitation researchers [1–4]. While recommendations have been made for mentoring this group of researchers [2], there is little information available regarding the actual mentoring experiences of rehabilitation researchers and how these might contribute to their development. The purpose of this study was to explore research mentoring experiences and perceived mentoring needs of aging and disability researchers at different career stages in one Canadian province.

### 1.1. Methods

Potential participants were identified from the Ontario Rehabilitation Research Advisory Network (ORRAN) Aging and disability theme researcher database. ORRAN is a provincially funded organization mandated to promote rehabilitation research in Ontario, Canada. At the time of the study, there were seven ORRAN theme groups. Each theme group maintained a list of researchers who had communicated their interest in the theme in some way (for example, by attending a workshop, requesting further information from theme leaders, etc.). This list contained each researcher's name, affiliation, and position. The authors reviewed the Aging and disability theme researcher database and separated the names into three categories: clinician-researchers, new investigators, and experienced investigators. Interviews were limited to Ontario because the research was conducted to inform the ORRAN Aging and disability theme group regarding members' mentoring experiences and perceived needs.

Within this Canadian context, “clinician-researchers” were health professionals employed by a health-care facility to carry out clinical work. However, their job descriptions also included research responsibilities, generally within their disciplines. Typically these individuals have graduate but not postgraduate training. Unlike the academic-based researchers interviewed in this study, the clinician-researchers could not be split into “new” and “experienced” researchers, as their level of preparation would not allow them to compete successfully for larger, funded projects, as would be expected of experienced investigators in this context. 

“New investigators” were individuals with post graduate training (minimally a PhD) who were employed by a university to carry out academic responsibilities of teaching, research, and service. These positions are known as “regular academic tenure stream appointments”, and individuals are typically hired as “assistant professors”. The dossiers of individuals in these positions are reviewed approximately 5 years post hiring. At this time, teaching and research productivity are evaluated, and a decision is made regarding their continued employment (i.e., whether they are granted tenure). With very few exceptions, individuals in these positions are not dependent on successful grant proposals for any part of their salaries. However, research productivity (including successful grant proposals) determines whether their positions become permanent.

“Experienced investigators” were individuals employed by a university who had been granted tenured and promoted to the rank of “associate professor”. Again, with very few exceptions these individuals are not dependent on successful grant proposals for their salaries. Typically, these individuals are eligible to be reviewed for promotion to the rank of “full professor” 10 years after hiring. Promotion to this senior rank is made on the basis of teaching and, particularly, research productivity.

In the current study, invitations to participate were sent by e-mail to ORRAN members identified as working in one of these three capacities (clinician-researcher, new investigator, and experienced investigator). A research assistant contacted all individuals who replied to the invitation and attempted to schedule focus group interview times to accommodate all or most of those interested in participating. Individuals who could not attend a focus group were invited to an individual interview. 

All focus groups and individual interviews were carried out by telephone to allow participation across regions. The interviews were tape recorded and transcribed verbatim. A semistructured interview guide was used. Individuals were asked to describe their experiences of mentoring, illustrate what had been positive or negative about these, explain their present mentoring needs, and describe any mentoring they themselves were providing. These interviews began with the question, “Think of a helpful mentoring relationship or mentorship experience that you currently have or have had to date in your career. What was it about this situation that made it helpful for you?” 

A generic qualitative approach was taken and as such, this study was “not guided by an explicit or established set of philosophic assumptions in the form of one of the known qualitative methodologies” [14, page 4] such as grounded theory or phenomenology. The focus of the study was on understanding the experience of mentoring for rehabilitation researchers. The verbatim transcripts were analyzed by one of the authors (Mary Egan) who summarized the content of the interviews and focus groups for each investigator group and then inductively identified themes of responses. The data was then re-examined for themes across the groups [15]. This analysis was reviewed by another author (Judy King) who had carried out the interviews. Focus group members were sent a draft copy of analysis and invited to provide feedback. 

The study was approved by the University of Ottawa Health Sciences and Sciences Research Ethics Board.

## 2. Results

Over half of the participants responded to our invitation to read this report, and all agreed that the analysis presented a good understanding of their experiences. 

Presentation of the results takes the following format: the content of the interviews with each of the researcher groups is first summarized according to the major questions addressed. Then the themes that arose from the interviews are described. 

### 2.1. Clinician-Researchers

Three of eight clinician-researchers contacted participated in the clinician-researcher focus group interview. These participants included two women and one man; their professional backgrounds were in nursing, occupational therapy, and therapeutic recreation. Two had completed graduate training. Each held a clinical position in a large teaching hospital. Each had both clinical and administrative responsibilities and expectations on the part of their employer that they would be involved in research. Two of the three clinician-researchers had had informal mentoring relationships, that is, a relationship where the mentor had not been formally assigned or recognized as a mentor. One participant had a mentor within the institution, and one had a mentor who was a university-affiliated researcher. Helpful mentorship relationships were characterized as being supportive of the mentees' learning and development, by providing practical help and encouragement to try new things. As one participant said

*…the trust that the mentor has in me and allowing me to take chances in my learning and giving me a little bit of a push to try something new, something outside of my comfort zone… They gave me feedback on how things were going, gave me suggestions with examples. I'm a hands on person so they gave me, “here's how it works”. There was lots of communication [without someone] hanging over my shoulder but just allowing me to try things, but then checking in with me.*

Another participant noted that the mentor's ability to challenge her was the most substantial aspect of the helpful mentoring experience. She also appreciated the mentor linking her with people who had needed expertise to help her pursue her research interests and development. 

In terms of present needs, all participants were particularly interesting in mentorship that would allow them to carry out research projects based on clinical questions they had identified, using data routinely collected during patient care *“to incorporate research into daily routine along with all the other commitments and looking at the aspect of taking something you're already doing, so not a make-work project, but taking all the outcome measures that you're using, all the information and making that into research and having something to show for it.”*

 The ideal mentor shared theoretical, substantive, and practical knowledge. Theoretical knowledge was important to determine how projects should be set up to ensure valid findings. Substantive knowledge was important to ensure linkage with relevant existing knowledge and experts. Practical knowledge was important for logistic aspects of the projects, such as when and how to get ethics approval, and how to set up a project so that it could be carried out within the clinical day.

Participants cited a number of mentorship resources, but none of these seemed to be easily accessible. Research and evaluation teams existed within their institutions but, due to limited resources, these teams were more involved with projects that had potential impact throughout the institution, not just within the participants' departments. University-based researchers were seen as a resource but were often more involved with student research projects being carried out at the institution rather than with participant-led projects. Professional practice leaders were also noted as a resource. (Within the Canadian context, rehabilitation professionals are often managed within diagnosis-based teams and report administratively to the team manager. However, they also have access to “professional practice leaders”—individuals who are appointed within the institution to provide guidance and coordination on discipline-specific issues). However, since the participants were either working in practice leader positions or in positions with similar responsibilities, these individuals were essentially their colleagues. 

None of their employers had a formal mentoring program that included researchers at their level. Participants were reluctant to approach potential mentors within or outside their own institutions. Small gatherings and events with the specific goal of bringing people together to work collaboratively made it easier to approach potential mentors. Other types of conferences were not seen as conducive to this, as academic researchers seemed to be busy catching up with other academics at these events. Working groups were sometimes struck during these conferences to address particular research issues, and these were seen as providing potential mentorship opportunities for clinician-researchers. However, these groups seemed difficult to sustain. 

All of the participants in this group noted that they had provided clinical and research mentoring to both staff and fieldwork students as part of the clinical leadership role they held. However, they also expressed difficulty in carrying out research mentoring, particularly due to limited knowledge and challenges motivating clinicians to consider taking part in research.

### 2.2. New Researchers

The new researcher focus group was made up of 5 of the 9 new researchers invited to attend. All were women. These researchers held faculty positions related to health studies, kinesiology, nursing, occupational therapy, and physical therapy. Four of the participants had at least one mentor for research. Two had informal mentors (including a former postdoctoral supervisor and a former doctoral committee member), and two had formally assigned mentors. 

At least two participants were surprised that more senior faculty did not provide mentoring to them when they began their academic positions. “*I've tried [linking for mentorship] with several colleagues that I thought I would be a good match content-wise when I started as a professor…I was so idealistic and naïve… I thought when new people come here the older colleagues embrace you and they invite you into their [grant proposals] and into their research projects… For the past two years… every grant I got I was PI [primary investigator] on…I did not participate on other peoples' [grant proposals]… I work in aging and I thought everybody who is working in aging should be one happy family.” *A second researcher agreed:* “It's not like that.*” 

To deal with this problem, the participants either applied for funding independently or with more senior colleagues as coinvestigators or sought help from investigators with expertise outside of their fields. Mentors with complementary expertise were seen as particularly helpful since this avoided problems with intellectual ownership of projects where junior mentee and senior mentor had overlapping expertise. It also avoided issues of new faculty being seen as potential competitors. 

In mentorship situations viewed as helpful, mentors had assisted mentees prepare for annual review and determine where to apply for research grants. These mentors also shared their experience with specific granting agencies, put unsuccessful ventures into perspective, assisted mentees obtain helpful positions (e.g., graduate advisor), and provided complementary expertise on grant proposals. Participants stated that mentors who let them examine their own previous successful grant proposals were particularly appreciated. The sharing of research granting agency feedback on unsuccessful submissions was also helpful. 

In addition to formal aspects of research, participants also noted they were grateful to mentors who could discuss work life balance within a research career. One participant stated “*I'm obviously struggling pre-tenure to put in the hours when I've got two young children, but I do. One of my mentors has children and we talk about that a lot and I think that's an important piece.”* Others seemed quite eager to speak with more senior researchers regarding how they had become successful: *“I just wondered if people [who] are successful … how did they how did they get everything done that they do in a week? Am I the only one that takes forever to write a grant [proposal], who looks at every single word again and again and again? Is that what it really takes? Are there any tricks to doing this any faster? …When does it become easier? Does it ever get easier?” *These feelings were echoed by another participant:* “I'm glad you're saying all these things. I'm not the only one.”*

One of the participants noted some difficulty with individuals who had mentored them as graduate students and with whom they were still involved with at the faculty level. This participant described that she had to be quite assertive in pointing out that she was now a colleague on a project rather than a research assistant. Another participant noted that she, herself, had difficulty adjusting to this new situation: “*You graduate and they expect the relationship to change and I say, “How am I supposed to be in this new relationship?*” While some mentors encouraged a more collegial relationship, others seemed to maintain aspects of the student-supervisor relationship. This put pressure on the mentee to perfect any work before it was brought before the mentor, which slowed progress. “*That feedback I got from one of my mentors [was], “What the heck is this? What happened?” Well I'm busy now! I'm teaching and I'm thinking that perhaps I can show you stuff in a rough draft format but I guess I cannot.”*

Knowing what they could ask from more senior researchers was also difficult. *“Every [research] grant I'm on I'm writing the whole thing almost and I wonder is it because I'm junior, do other people when they have co-investigators, do they do any work on the grant [proposal]? And I wonder am I still in the junior role?...I feel like I can ask them their opinion but I do not feel I could ask them to write a section [of a grant proposal].”*

Other participants noted that their mentors did not seem to understand how busy they were with teaching responsibilities. One participant recounted how her mentors helped her strategize when she pointed out to them that the demands of teaching and starting up a research program were overwhelming her. *“They started strategizing with me…trying to help me to apply for any opportunities… that would involve course release. So that's what we're focusing on now.”* Other participants noted the difficulty balancing teaching and research and that they had been able to restructure their timetables to make this somewhat more manageable with help from the program chair. However, all participants had to first approach the chair and suggest ways that their teaching could be better scheduled. 

Participants appeared very eager to provide mentorship to others, and a number of them were actively engaged. One said, *“… doctoral students come to talk to me about their transitions that are pending and [there are] four of them and we meet informally. We go for sushi [and] they ask me all the things about interviews, applying for jobs, post-docs, how did I make my decisions.*” But some participants were also struggling to figure out how to carry out this new role. *“I thought that the other day, “Am I starting to mentor this person?” Because she's asking, she invited me to be on her grant [proposal] and she's asking me questions and it's kind of weird because I'm not used to that kind of role…” *Another researcher echoed this sentiment:* “ I'm writing a paper with a student and now I'm the one that's supposed to help get this paper ready for publication and it's kind of a weird place because it's of the level of uncertainty …whether the paper is ready or not.”*

### 2.3. Senior Researchers

Of the 17 senior researchers contacted, 8 agreed to participate. Five senior researchers attended two focus groups, and three were interviewed individually due to time conflicts. The senior researcher participants were four men and four women. Their backgrounds were in nursing, occupational therapy, physiotherapy, speech-language pathology, rehabilitation engineering, and medicine. Seven of the senior researcher participants identified that they had had at least one formal mentor. These mentors came from graduate, doctoral, and postdoctoral supervisors, mentors formally identified as a requirement of personnel awards, and senior researchers within a university or research institute. 

For these participants, early in their careers important mentors asked the big questions, such as *“Why does this interest you? Where do you want to be in the future?”* These mentors helped the participants envision themselves as future successful researchers. They “opened doors” for them, ensuring they met important individuals and got involved in projects where they could develop skills and networks. Helpful mentors demonstrated and encouraged meeting deadlines and routinely presenting and publishing work. They also assisted the developing researchers in learning to balance, and valuing balance, between work and family life. As well, these mentors cushioned the blows inherent in academic work by putting these into perspective. 

Some of the participants noted that they had had colleague mentors. These were mentors from different but related fields; they helped the participants ensure that their work would have relevance to other clinical fields, exposed the participants to important ideas within their fields, and provided fresh critiques of work based on their different perspectives.

A number of participants noted an absence of mentoring at points they perceived mentoring could have been extremely helpful. One researcher noted that when he came to his research position, having trained outside of Canada, he had no one to translate the research granting system to him which resulted in substantial inefficiency: “*I did not understand that you could go for renewal… so for my first five or ten years I was not renewing [grant proposals] when at a time when the probability of renewal was … twice that of applying for the new grant.”* Another participant noted the absence of mentoring when she was a PhD student which was detrimental for her early career development: “*I'd say for the first few years, no one helped us with publications, they never even thought that perhaps we'd help out on a grant [proposal]… Very much it was slugging away on your own …What happens … is [now research grant reviewers] are looking for post doc publications, [and students in my cohort] do not have any publications because we had a model where you were not included.” *This participant felt that this reflected the newness of the PhD program she was in; no one seemed to realize the importance of mentoring PhD students or how to do it. 

Other participants spoke of mentoring experiences they had perceived as unhelpful. One participant spoke of a mentor's negative views regarding the impossibility of combining research and family life. This led to an atmosphere that was somewhat hostile to her. Another participant talked about a mentor who set unattainable benchmarks, making performance reviews very discouraging. Two participants noted that lack of specific feedback on performance was associated with disappointing mentoring experiences. 

Most of the participants spoke about specific, current mentoring needs. One noted that she required mentoring for developing grant proposals that required methodologies that she had not previously used. This researcher also intimated that it would be good to have some way of participating in discussions that may affect the direction of Canadian rehabilitation research (for example, what is the place of randomized controlled trials in Canada where funding for these is quite limited). Three others related desiring mentorship regarding administrative aspects of research, such as directing a team: “*I'm really at a place where I need to be doing the bigger group projects, like team [research] grant types of things, and that's a set of management skills that is different than just having a simple operating grant,… managing those kinds of things … even just the ins and outs of research finances and human resource systems in the way we work.”* Personnel issues included developing skills in managing independent researchers working under their direction. 

All of the senior researcher participants were very involved in mentoring. A number of participants appeared to have reached a turning point where they felt a definite responsibility to assist newer researchers, although one that had to be balanced with their own productivity criteria: *“I'm actually finding one of my own struggles is, particularly given how competitive funding is right now, I need to be mentoring and giving junior people the opportunity for the leg up as opposed to that it's always got to be me, so it's a balance because I mean I cannot completely do that because obviously I have to have a track record myself to keep a job but to me there is a really big mind shift that should be happening.”*

Most of the senior researcher participants were currently providing mentorship through research teams, although at least four of the researchers met with trainees regularly on an individual basis. These researchers set aside regular meetings times for trainees and believed that these scheduled appointments helped trainees appreciate that they were available for them; as well, these meetings seemed to help trainees learn to organize their projects and keep their work on track. 

The researchers who valued the group mentoring model did so for a number of reasons. First, it was seen as an efficient model, where all, from graduate students to senior researchers, could learn from each other. Second, it decreased the potential intensity of the mentoring relationship and assured shared responsibility for mentoring. Third, it allowed student-to-student relationships to develop, which were seen as important for some aspects of academic life. While all participants felt that face-to-face interaction was necessary, two felt that mentoring could be done at distance if a relationship had first been developed face to face. 

### 2.4. Mentoring for Transitions

The overarching theme identified from the data was “*mentoring for transition*”. Participants across career stages referred to positive mentoring experiences as those that helped them transition either from their previous stage into the present stage or from the present stage into their next career progression. Unhelpful mentoring experiences were characterized by actions that seemed to hinder transitions. 

Helpful mentoring could begin during professional and/or undergraduate studies when students were introduced to the idea that their future careers may include research. At the doctoral level individuals were challenged to envision their academic research program by identifying where their skills and passion lay. New investigators were aided by mentors who helped them reflect on work-life balance and how to take failure in stride. Established investigators were seeking mentoring for assuming a leadership role within their fields. 

Three categories were subsumed under this theme. The first two related to the type of knowledge that mentors helped participants develop. The first “*hidden information*” related to information that is critical to the process of carrying out research but can be difficult to access (e.g., procedures for obtaining relevant ethics board approval). “Hidden information” also referred to information only available to those with specific research experience in the area (e.g., how best to frame a grant proposal for a particular agency). 

The second category, “*delicate issues*”, referred to knowledge that the participant felt that perhaps they *should* have or that discussion of which could put them in a vulnerable position. This included questions related to juggling teaching and research responsibilities, work-life balance, dealing with rejection of grants proposals and papers, knowing what to expect from more senior colleagues in terms of their contributions to grant proposals and papers, and managing relationships within a team. Mentors who reassured participants that these were concerns they had struggled with, and who shared their personal experience, were seen as extremely helpful. 

The third category, “*special challenges of clinician-researchers*”, related to this group of investigators uniquely. Unlike the other two groups, these participants had one transition only, and that was the transition from clinician to clinician-researcher. Particularly to this group as well, it appeared that there were no other researchers who were now beyond this transition and could provide the clinician-researchers with hidden information and discuss delicate issues with them. While academic researchers helped at least one of these participants to envision herself as a researcher and seek out new, necessary skills, these mentors seemed generally difficult to access. As well, the “delicate issue” of most interest to this group (how to use clinical data to carry out research that can be fit within a clinical day) was perhaps something that academic researchers may not have experience with. With no clearly delineated career progression or mentorship from those who had been there before, this group really appeared to be struggling. 

## 3. Discussion

Our participants confirmed the importance of mentoring to their research and career progress. Their descriptions of helpful mentoring experiences reflected both career and psychosocial aspects of mentoring [6] as well as de Janasz and Sullivan's [7] categories of assistance with issues of “why”, “how”, and “who” that span the career trajectory. Unlike de Janasz and Sullivan, however, we found that issues related to work-life balance crossed all career stages. Interestingly, these issues were raised by both male and female researchers. 

Participants described valued mentoring that helped them make the transition from one career stage to the next. Classic definitions of mentoring stress that the process leads to development of the mentee, implying transition. Our findings highlight what participants viewed as critical to their successful transition at each stage of the typical academic career path. For example, issues related to balancing different demands on their time and establishing themselves as independent investigators were foremost in the discussions of the new investigators. Issues related to leadership roles seemed to be the principle concerns of experienced investigators. 

Interestingly, within the experience of the participants, often it appeared that when mentoring was seen as unhelpful it was because the mentor did not recognize that an important transition was occurring (for example, from student to professor and colleague) or was highly pessimistic regarding the mentee's chances of succeeding given such things as present performance or family commitments. In contrast, within helpful mentoring relationships mentees were assisted to envision their futures as successful researchers and from there plan necessary steps towards this future.

All of the groups expressed needs for assistance with “hidden information” and “delicate issues”. Researchers were particularly grateful to mentors who volunteered to help with the latter without having to be directly asked, as there was some feeling on the part of the participants that questions regarding these issues could reflect poorly on their abilities as researchers. 

The experiences of our participants reflected some notable mentoring deficiencies among the rehabilitation researchers we interviewed. Participants who worked outside of a regular academic appointment seemed in many ways at the greatest disadvantage. These individuals were attempting to manage one career transition (from clinician to clinician-researcher) with few mentoring resources. Their desire to carry out research using the clinical data they had available seemed motivated by the limited time that they had available to devote to research, but it is not clear whether this type of research was feasible or would help them address the questions of treatment efficacy which were of interest to them. While participants perceived that helpful mentorship would facilitate this type of research, ultimately these individuals may benefit more from assistance defining their positions and goals.

Among those on a traditional academic path, what was most concerning perhaps was the experience of new faculty who found themselves quite alone in their early career development. Rather than having senior mentors who could provide opportunities for learning while participating in collaborative research and developing their curriculum vitae, these individuals were left to develop grant proposals on their own in a highly competitive environment. The experience of being left to “sink or swim” pretenure was evident in the early careers of both the junior and senior researchers we interviewed. This latter tendency has been criticized due to the human and economic cost of not providing adequate support to launch individuals in their academic careers [7]. 

Some junior researchers found senior faculty open to participating on their grant proposals. However, they were unsure what they could ask of these senior researchers. The junior researchers appeared to be asking for help understanding the hidden information and delicate issues surrounding how a junior faculty member should act when collaborating with a more senior faculty member.

For some new researchers who had mentors, the mere presence of a mentor did not ensure a helpful mentoring experience. Notably, mentors who had previously supervised the researcher could present difficulties when the mentors continued to treat the new researchers as students (for example, some mentors had difficulty seeing their work with the new researcher as a collaborative venture rather than an assignment to be evaluated). It seems that both junior researchers and senior researchers could benefit from mentoring concerning issues related to evolving relationships.

Formal mentoring programs have certainly demonstrated success in academia [6]. However, these programs require resources and administrative commitment [8, 9] and can be difficult to sustain [10–12]. Many of the participants spoke of mentoring that occurred informally within research collaborations. For them, what they considered mentoring did not always require a more senior person working with a junior person. For example, new investigators spoke about being mentored while collaborating with investigators with complementary expertise. To achieve some of the benefits of mentoring without launching formal mentoring programs, institutions may wish to reinforce these mentoring opportunities. For example, in institutions where new investigators may be seen as competitors within a field, more formalized opportunities for cross-department collaboration may provide helpful mentoring opportunities. In addition, senior faculty could be reminded that junior faculty may appreciate their opening discussions on hidden information and delicate issues. It may be important, however, to monitor issues that may arise when the mentor and mentee have another relationship as well (such as supervisor-supervisee or colleague-colleague) with its own expectations and responsibilities.

It is important to note that these findings represent the participants' self-perceived mentoring experiences and needs. Whether more of the types of mentoring that they perceived as helpful at particular points in their careers may have made a difference to their research success is not possible to determine. As well, approximately one half of individuals approached did not volunteer to participate in this study. It is possible that at least some nonparticipants may have had very different mentoring experiences or may have found mentoring irrelevant to their work. Moreover, at least some others may have been reluctant to share their experiences of this potentially sensitive topic in a group setting. In addition, while all participants were involved in rehabilitation research concerning disability and aging, these individuals formed a relatively heterogeneous group, with varying professional and academic backgrounds. It is possible that the themes uncovered were idiosyncratic to this group, rather than transferrable to rehabilitation researchers in general. As well, transferability of our results is limited by the preponderance of women in the new investigator group in addition to the fact that our sample was relatively small and restricted to one Canadian province.

However, our methods allowed for a relatively in-depth discussion with these rehabilitation researchers. Particularly in the focus groups, participants offered candid examples and opinions when it became clear that they were not alone in their experiences. Member checking revealed that participants felt that the analysis presented a good understanding of their experiences. 

Our findings indicate that rehabilitation researchers at all career levels may be interested in mentoring to help them with career transitions. Helpful mentoring for rehabilitation researchers seems to assist with career transitions by providing access to hidden information and help handling delicate issues, including dealing with rejection of publications and grant proposals and striving towards work-life balance. 
